# The Notch signaling network in muscle stem cells during development, homeostasis, and disease

**DOI:** 10.1186/s13395-022-00293-w

**Published:** 2022-04-22

**Authors:** Stamatia Gioftsidi, Frederic Relaix, Philippos Mourikis

**Affiliations:** 1 Université Paris Est Créteil, Institut National de la Santé et de la Recherche Médicale (INSERM), Mondor Institute for Biomedical Research (IMRB), F-94010 Créteil, France; 2 EnvA, IMRB, F-94700 Maisons-Alfort, France; 3 Etablissement Français du Sang (EFS), IMRB, F-94010 Creteil, France; 4 Assistance Publique–Hôpitaux de Paris, Hopital Mondor, Service d’Histologie, F-94010 Creteil, France

**Keywords:** Myogenesis, Notch signaling, Muscle stem cells, Myopathies

## Abstract

Skeletal muscle stem cells have a central role in muscle growth and regeneration. They reside as quiescent cells in resting muscle and in response to damage they transiently amplify and fuse to produce new myofibers or self-renew to replenish the stem cell pool. A signaling pathway that is critical in the regulation of all these processes is Notch. Despite the major differences in the anatomical and cellular niches between the embryonic myotome, the adult sarcolemma/basement-membrane interphase, and the regenerating muscle, Notch signaling has evolved to support the context-specific requirements of the muscle cells. In this review, we discuss the diverse ways by which Notch signaling factors and other modifying partners are operating during the lifetime of muscle stem cells to establish an adaptive dynamic network.

## Background

**Fig. 1:**
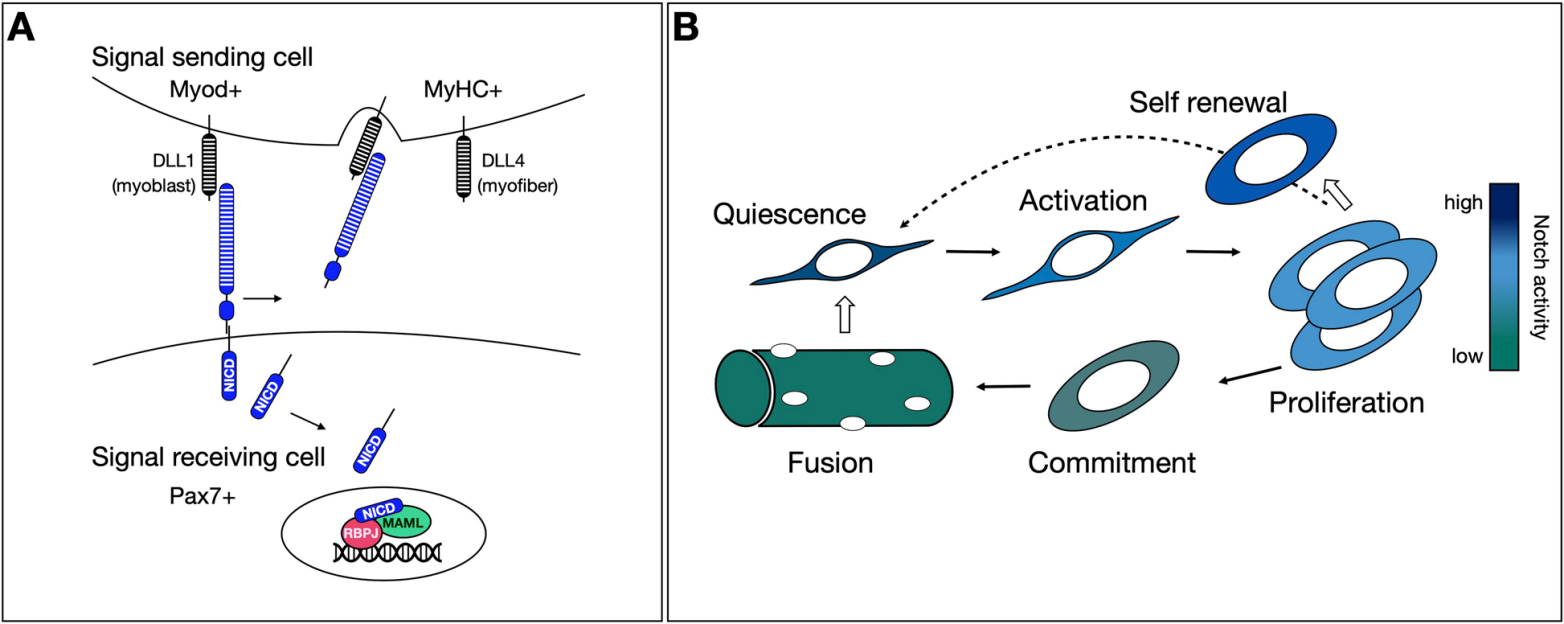
The Notch signaling pathway during myogenic progression and self-renewal. **A** Basic scheme of the Notch signaling pathway in murine muscle cells. The receptor is more highly expressed on the stem/progenitor cell (signal receiving cell), whereas the DLL ligands on the committed myoblasts and the mature myofibers (signal sending cells). Ligand-receptor interaction triggers intramembrane proteolysis and release of the intracellular domain of Notch (NICD). NICD then translocates into the nucleus where it forms a complex with its main downstream effector and DNA binding transcription factor RBPJ, and members of the coactivator Mastermind-like (MAML) family. The triprotein NICD transcriptional complex recruits additional coactivators and histone modifiers to activate transcription, not illustrated here for simplicity. **B** During MuSC activation and myogenic commitment, Notch signaling activity is downregulated. Quiescent MuSCs have high Notch activity (dark blue in color key), which maintains *Pax7* and inhibits *Myod* and *Myogenin* expression. Immediately after MuSC activation, Notch activity rapidly declines and the cells express MYOD, which accelerates S-phase entry. During the proliferation phase, high Notch activity is restricted to some cells, which remain undifferentiated and self-renew to replenish the satellite cell pool (dotted arrow). Notch activation is principally triggered by ligand-bearing differentiating myoblasts (block arrow indicates direction of Notch signaling). Mature myofibers, in which Notch activity is insignificant (green color in color key), are the main source of ligand in the resting muscle and maintain MuSC quiescence by direct cell-cell interaction (block arrow)

With the notable exception of blastocyst morphogenesis and the establishment of the three germ layers (ectoderm, endoderm, and mesoderm) [1], Notch signaling is involved in the formation of virtually every tissue studied to date and, not surprisingly, has emerged as a major regulator of stem cell functions. Notch is a highly conserved transmembrane plasma receptor that mediates cell-cell communication. Similar to the murine Notch receptors (Notch-1, -2, -3 -4 in mammals), its ligands (Delta-like (DLL) -1, -4 and Jagged (JAG)-1, -2 in mammals) are also transmembrane proteins, so physical cell-to-cell interaction is required for activation of the pathway (note that *cis* receptor/ligand interactions are predominantly inhibitory [2, 3] and secreted, cleaved ligands seem to have no activity in vivo [4, 5]). Notch is one of a handful of plasma membrane receptors that acts both as a cell surface receptor and a transcription factor, together with the leukocyte-common antigen-related receptor tyrosine phosphatase (LAR), the amyloid precursor protein, and the receptor tyrosine kinase receptor ERBB4 [6, 7]. Following ligand-triggered intramembrane proteolysis, the intracellular domain of Notch (NICD) is cleaved and translocates into the nucleus, where it acts as a transcriptional coactivator [8, 9]. There NICD forms a complex with CSL (RBPJ in vertebrates, Su(H) in flies, Lag-1 in roundworms) and stabilizes its binding to DNA to induce gene expression [10–12] (Fig. 1A).

Muscle stem cells (MuSCs) depend on Notch signaling activity from their emergence in the embryo to their residence in adult tissue, both in resting and regenerating conditions. Embryonic and adult MuSCs share several common features, including the expression of the paired-box homeodomain transcription factor PAX7, and execute an almost indistinguishable myogenic program, involving the sequential expression of specific muscle regulatory factors [13]. A prominent difference, however, between adult and embryonic MuSCs is the anatomy of their cellular niche, which is strictly defined by the myofiber and the basal lamina in adult MuSCs but is highly disorganized in the growing muscle. Another distinct difference is the proliferation of Pax7^+^ cells during murine muscle growth, whereas in adult tissue, MuSCs are mitotically quiescent. Regardless of the developmental stage of MuSCs, Notch signaling has been shown to be indispensable for both of these contrasting MuSC states. Both embryonic and adult MuSCs with abrogated Notch signaling ectopically differentiate, leading to a depletion of MuSCs and progenitors whereas in the context of growing tissue, Notch signaling mutations lead to sarcopenia [14–21]. In reciprocal experiments, overexpression of constitutively activated NICD, terminal myogenic differentiation was blocked, leading to a loss of muscle mass; a phenotype akin to the abrogation of the pathway [22–25]. In Table 1 we summarize the Notch signaling mouse models that have been reported with a muscle phenotype. Of note, the role of Notch signaling on muscle stem cell homeostasis and regeneration is conserved in other vertebrates, including the zebrafish [41] and the chick [42, 43].

**Table 1:** Notch signaling mouse mutants with MuSC phenotype

Gene	Mouse model	Phenotype of MuSCs	Reference
**Core Notch members**			
Rbpj	*Tg:Pax7-CreERT2*; *Rbpj*^*flox/-*^	Spontaneous differentiation, bypassing S-phase. Reduction of quiescent pool starting at 16 days post-tamoxifen induction. Failure to self-renew.	[18]
Rbpj	*Pax7*^*CreER*^; *Rbpj*^*flox/flox*^	*idem*	[14]
Rbpj	*Lbx1*^*Cre*^; *Rbpj* ^*flox/flox*^	Precocious differentiation, muscle hypotrophy.	[20]
Rbpj; Myod dKO	*Pax3*^*Cre/+*^; *Rbpj*^*flox/+*^; *Myod*^*−/−*^	Rescued differentiation, disrupted homing under the basal lamina.	[26]
Notch1	*Pax7*^*CreERT2/+*^; *Notch1*^*flox/ flox*^ (post-natal day P7 induction)	Failure to enter quiescence at 4 weeks of age, differentiation, and eventual reduction of pool size.	[27]
Notch1	*Pax7*^*CreERT2/+*^; *Notch1*^*flox/ flox*^ (adult induction, 7-14w old)	No phenotype up to 19 days post-tamoxifen induction.	[28]
Notch2	*Pax7*^*CreERT2/+*^; *Notch2* ^*flox/flox*^ (adult induction, 7-14w old)	Reduction of pool at 5 days post-tamoxifen induction.	[28]
Notch1/Notch2	*Pax7*^*CreERT2/+*^; *Notch1*^*flox/flox*^*; Notch2* ^*flox/flox*^ (adult induction, 7-14w old)	Drastic reduction of pool 5 days post-tamoxifen induction.	[28]
*Notch3*	Germline *Notch3−/−*	Increased size of quiescent pool, increased proliferation in culture. Muscle hyperplasia following repetitive muscle injuries.	[29]
N1-ICD O/E	*Pax7*^*CreERT2/+*^; *R26*^*NICD-nGFP*^ (post-natal day P7 induction)	Enter premature quiescence 3 days post-tamoxifen induction	[27]
N1-ICD O/E	*Myf5*^*Cre/+*^; *R26*^*NICD-nGFP*^	Inhibition of differentiation and sustained proliferation at embryonic stages. Enter premature quiescence at fetal stages. Muscle hypotrophy.	[23]
N1-ICD O/E	*Pax7*^*CreER*^; *R26*^*NICD-nGFP x*^	Upregulation of Pax7 independently of Myod inhibition. Inhibition of quiescence exit following isolation.	[30]
Dll1	*Dll1*^*hypomorphic*^*/Dll*^*null*^	Precocious differentiation, muscle hypotrophy.	[19]
Dll1	*Pax7*^*CreERT2*^; *Dll1*^*flox//flox*^	No impact on quiescence or activation. Premature differentiation, impaired self-renewal, and myofiber diameter severely reduced upon regeneration.	[31]
Dll4	*HSA*^*CreMER*^; *Dll4* ^*flox/flox*^	Reduction of quiescent pool.	[32]
Dll4	*Pax7*^*CreERT2*^; *Dll4* ^*flox/flox*^	No impact on quiescence.	[32]
**Notch targets**			
Hey1/HeyL co-dKO	*Pax7*^*CreERT2/+*^; *Hey1*^*flox/flox*^; *HeyL*^*–/–*^	Pool size reduced 3 weeks post-tamoxifen injection. Reduced weight of regenerated muscle and increased fibrosis.	[33]
Hes1	*Pax3*^*Cre/+*^; *Hes1*^*flox/flox*^	Reduced pool size at post-natal day 28. Subtle effect on the overall muscle size at birth, severely affected muscle growth during post-natal development.	[34]
Hes1/HeyL	*Pax3*^*Cre/+*^; *Hes1*^*flox/flox*^; *HeyL−/−*	Greater reduction of pool size compared to single coHes1.	[34]
Col5a1	*Tg:Pax7-CreERT2*; *Col5a1*^*flox/flox*^	Spontaneous differentiation, reduction of quiescent pool starting at 3 weeks post-tamoxifen induction. Failure to self-renew.	[35]
mircroRNA-708	WT (injected antagomir)	Spontaneous migration and differentiation by targeting *Tensin3* transcripts.	[36]
**Notch modifiers**			
Pten	*Pax7*^*CreERT2/+*^; *Pten* ^*flox/flox*^	Spontaneous activation of quiescent MuSCs and premature differentiation without proliferation (reach S-phase but seem not to complete the cell cycle). Failure to self-renew.	[37]
Mettl3	*Pax7*^*CreERT2*^; *Mettl3*^*flox/flox*^	No impact on quiescence. Inhibition of proliferation, impaired regeneration.	[38]
Adam10	*Pax7*^*CreERT2/+*^; *Adam10* ^*flox/flox*^	Reduction of quiescent pool, regeneration defect.	[39]
Foxo3	*Pax7*^*CreER*^; *Foxo3* ^*flox/flox*^	Impaired self-renewal and increased propensity to differentiate.	[40]

Effectively, a major function of Notch signaling in skeletal muscle is to sustain an upstream population of founder cells irrespective of their cycling status by safeguarding their undifferentiated state (Fig. 1B). More recent studies have elucidated the *modus operandi* of Notch pathway members, providing mechanistic explanations of their antimyogenic activity, their crosstalk with other signaling molecules and the interactions between heterologous cell types. In this review, we will provide an updated view on the ways by which Notch signaling factors and other modifying partners are operating during the establishment, maintenance, and self-renewal of MuSCs.

### The core antimyogenic activity of notch decoded

**Fig. 2:**
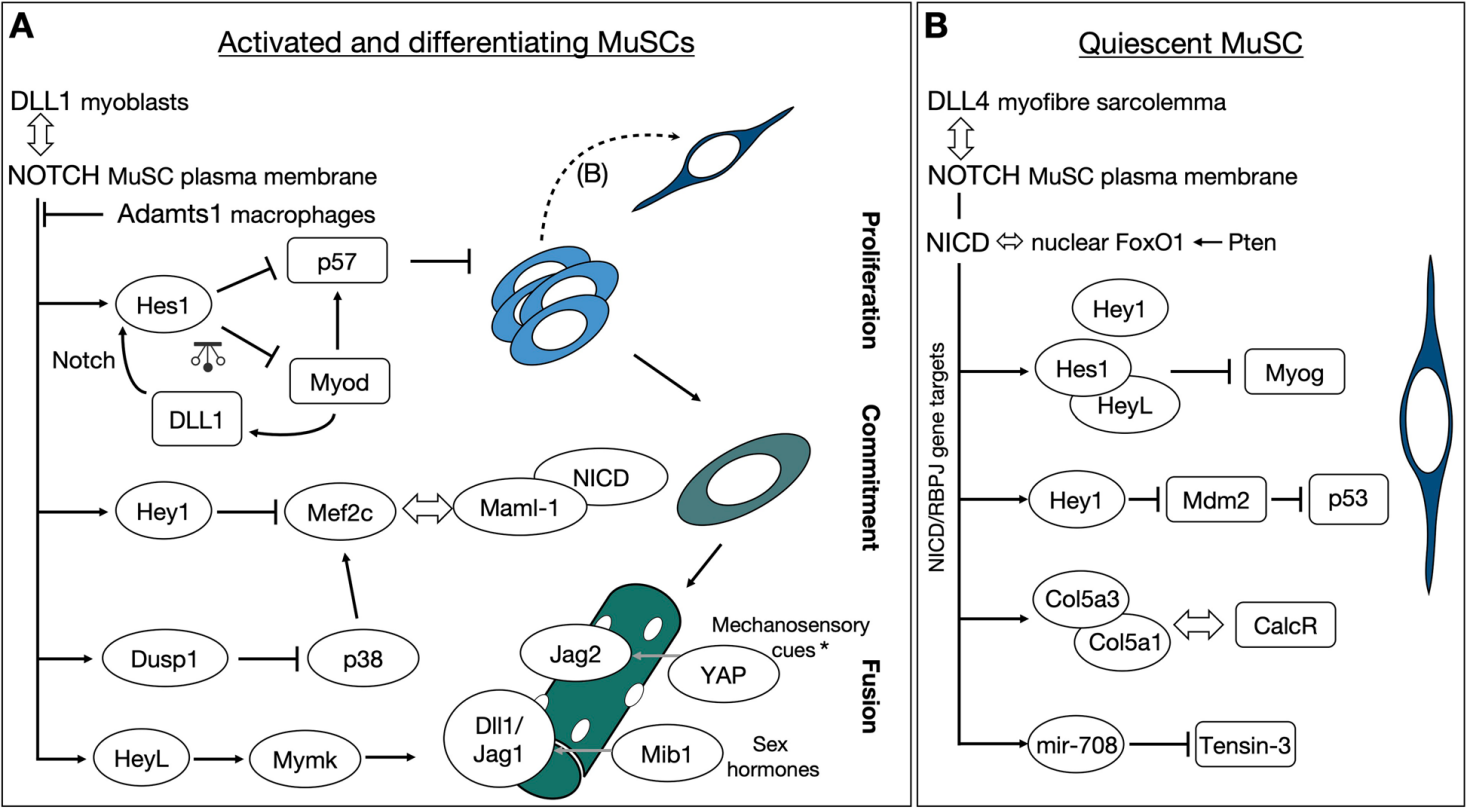
The Notch signaling network in murine muscle stem cells. **A** In the proliferating cells, enhancer competition and negative autoregulation establish an oscillatory system (pendulum sign) comprising transcription factors and ligands that regulate each other. Competition is also occurring for the transcriptional co-activator Mastermind-like 1 between the NICD activating complex and the differentiation factor Mef2c, while Mef2c is under the control of the Notch-controlled Dusp1 kinase that targets p38. Notch signaling also safeguards cells from spontaneous fusing by repressing the expression of the membrane activator of myoblast fusion Myomaker (Mymk). Ligand presentation on the growing fibers is stimulated by extrinsic cues, including mechanical stress (fetal chick fibers) and circulating sex hormones (puberty and muscle regeneration). The factors that maintain quiescence (Fig. 2B) are reiterated for self-renewal. Double-headed arrow indicates protein interaction; pendulum sign indicates oscillation; dashed arrow line indicates self-renewal; *the YAP/Jag2 link has been demonstrated in chick embryos. **B** Notch signaling maintains both quiescent and activated MuSCs by engaging different targets and interactors. Quiescent MuSC express Notch-1, -2, and -3 and, in the mouse, the principal ligand is Dll4 from the myofibers. Diverse direct NICD/RBPJ transcriptional targets execute different functions: the Hes/Hey repressors prevent the expression of differentiation factors, collagen V chain encoding genes directly contribute to the build-up of the quiescent niche, and micro-RNA mir-708 anchors MuSCs by targeting molecules involved in cell migration

The inhibition of differentiation by Notch signaling is principally driven by the well-characterized and highly conserved direct targets of the pathway, the basic-helix-loop-helix transcriptional repressors of the Hes/Hey family. In mouse skeletal muscle cells, the strongest responders to Notch activation amongst them (in order of transcriptional fold-induction) are *HeyL*, *Hey1*, and *Hes1* [16, 23, 44], whereas in human myoblasts *HES1* seems to be the most highly induced [11, 45]. Overexpression, though, of *HeyL* or *Hes1* alone in the immortalized myogenic cell line C2C12 is not sufficient to block myogenesis [46]. It is the HEY1 repressor that does so by being recruited to the promoters of *Myogenin* and myocyte enhancer factor 2C (*Mef2C*) genes, whose products are critical for muscle differentiation [44]. Surprisingly, *Hey1* germline knockout mice do not show MuSCs abnormalities [16]. Similarly, the number of adult MuSCs is mildly decreased or not affected at embryonic day (E) 17.5 in the hindlimb muscle of *HeyL* germline knockout mice [16]. Double-knock out *Hey1/HeyL* mice on the other hand, exhibit severe regenerative defects due to a reduction in MuSC number, resulting from increased *Myod* and *Myogenin* expression [16]. More recently, it was shown that HEYL forms heterodimeric complexes with HES1 and acts synergistically to bind with higher affinity to the *Myogenin* promoter [33] (Fig. 2A). Consistently, the removal of *HeyL* in *Hes1* null cells (*Pax3*^*Cre*^; *Hes1*^*flox/flox*^; *HeyL−/−*) exacerbates the MuSC deficiency observed in *Pax3*^*Cre*^; *Hes1*^*flox/flox*^ single mutant muscles [34].

The redundancy observed between the different HES/HEY proteins is not conserved in the context of muscle hypertrophy. Following overload-induced hypertrophy, the majority of MuSCs gets activated but shows some distinct features compared to the classical activation that is described in regenerating muscle and *HeyL*, unlike *Hey1*, is not downregulated upon activation. In fact, HeyL is required for the proliferation of MuSCs during induced hypertrophy, contrary to muscle regeneration [47].

Notch signaling seems to have evolved several safety lock mechanisms in muscle cells to prevent ectopic differentiation from occurring and to enhance regulation of transition states. In addition to the direct transcriptional repression of *Mef2C* by HEY1, a fine balancing mechanism has been uncovered whereby the essential transcriptional NICD coactivator, Mastermind-like 1 (MAML-1), is bound and sequestered by the MEF2C protein [48]. In C2C12 cells, Notch induces the expression of *Dusp1*, a dual-specificity MAPK phosphatase that blocks the activity of the MAPK family member p38 [49]. This prevents p38 from phosphorylating and activating MEF2C and E47, which would usually drive myogenesis and promote MYOD/E47 association [49, 50]. An unexpected function of HEYL in myoblast fusion has more recently been uncovered, unravelling another safety lock of the Notch system. Since Notch inhibition drives early differentiation, the direct regulation on fusion had been overlooked. By analyzing chick embryonic myogenesis, Esteves and colleagues discovered that Notch inhibition increased the expression of TMEM8C, the chick orthologue of the fusion master gene *Myomaker*. Specifically, HEYL was found to bind and repress the regulatory regions of TMEM8C, thus hindering myoblast fusion [51] (Fig. 2A).

At the level of receptors, using tamoxifen-inducible MuSC-specific knockout mice for *Notch1* and *Notch2*, it was shown that the size of the adult satellite cell population is slightly reduced in *Notch2*-cKO, not significantly affected in *Notch1*-cKO, but almost completely depleted in double *Notch1/Notch2* knockout mice [28]. The functional redundancy between NOTCH 1 and 2 during muscle regeneration was later confirmed with neutralizing antibodies [52]. Germline *Notch3* knockout mice however, had an abnormally high number of MuSCs, pointing to an antagonistic function when compared with the other Notch receptors [29]. Several reports have compared NOTCH1 and NOTCH3 functions in different contexts and concluded that these Notch paralogues display distinct roles in stem cells. In neural stem cells of the zebrafish pallium, Notch3 maintains quiescence, in striking contrast from the role of Notch1 (Notch1b in zebrafish) that prevents differentiation of activated progenitors [53]. A similar functional dichotomy is observed in murine MuSCs, whereby *Notch3* knock out leads to an increase in quiescent MuSCs and accelerated proliferation of activated MuSCs on isolated muscle fibers [29]. It remains unclear and controversial on how NOTCH3 operates mechanistically to antagonize Notch signaling. In muscle cells, one hypothesis examines the possibility of NOTCH3 induction of *Nrarp* functioning as a negative feedback regulator of Notch signaling by attenuating NICD-mediated transcription [54]. The loss of NOTCH3 during muscle growth and regeneration could lead to over-stimulation of Notch signaling by *Nrarp* downregulation which could in turn partially explain the increased number of PAX7 cells, and thus the muscle hyperplasia in *Notch3* KO mice. Despite these hypotheses, further studies using conditional MuSC-targeted deletion of *Notch3* would be required to acquire more precise information on the role of this locus during MuSC growth and homeostasis.

### Dynamics and source of notch ligands in growing and resting muscle

The origin of MuSCs in the mouse trunk can be traced back to mesodermal cells of the dermomyotome [55], a transient epithelial structure in the somites formed in the mouse around embryonic day (E) 9. The muscle founder cells are initially marked by the transcription factors PAX3 and later by both PAX3 and PAX7 [56–58]. Their emergence in the dermomyotome does not seem to require Notch signaling, since they are unaffected in Notch mutant embryos [19, 20]. Like the mammalian central nervous system, Notch signaling is essential for maintenance but not the generation of neural stem cells [59]. This is consistent with the expression pattern of the Notch receptors -1, -2 and -3, and the ligands *Jag2* and *Dll-1* in embryonic MuSCs, which are excluded from the dermomyotome [RNA *in situ* hybridization [19];)]. The expression of Notch ligands is instead confined to the underlying myotome, where differentiating muscle cells reside and where Notch activity is critical. In *Dll-1* and *Rbpj* (*Pax3*^*Cre*^; *Rbpj*^*flox/flox*^ and *Pax7*^*CreERT2*^; *Rbpj*^*flox/flox*^) mutant mouse embryos, severe muscle hypotrophy has been observed due to precocious differentiation of the self-renewing MuSC population in the myotome [19, 20]; our unpublished data). Notably, the observation that the muscle phenotype in embryos with globally reduced activity of DLL1 resembles the *Rbpj* null MuSCs, strongly suggests that the prevalent Notch ligand in embryonic myogenesis is DLL1.

A similar scenario, with the same actors, seems to be played during regeneration in adult muscles. Using paralogue-specific antagonizing antibodies, it was shown that blocking DLL1 4 days after cardiotoxin-induced injury led to severe self-renewal defects. Using anti-DLL4, -JAG1 or -JAG2 during regeneration, instead, gave no regeneration phenotype [52]. The data produced by those two studies is comprehensive and has established that during embryonic myogenesis and adult muscle regeneration the essential Notch ligand is DLL1, whose function is not compensated by either DLL4 or JAG-1/2. However, in both studies, cells were targeted indiscriminately, thus preventing a precise pinpoint to the actual signal-sending cell type. In fact, both embryonic myogenesis and adult muscle regeneration involve extensive cell migration and mixing of MuSCs with diverse cell types [60, 61]. Mesenchymal cells, connective tissue fibroblasts, endothelial cells (ECs), and inflammatory cells from regenerating tissue, all constitute potential Notch signaling activators. This complex cellular system was genetically dissected using *Pax7*^*CreERT2*^; *Dll1*^*flox/fl*ox^ mice [31]. This mutation did not affect the quiescent MuSCs, and activation occurred accordingly as was observed using α-DLL1 neutralizing antibodies [52]. However, at the later stages of regeneration, the cells differentiated prematurely rendering decreased PAX7^+^ and increased MYOG^+^ cells at 4 dpi, and even fewer PAX7^+^ cells at 7 dpi and 21 dpi [31]. Importantly, this phenotype was recapitulated on isolated myofibers from *Pax7*^*CreERT2*^*; Dll1*^*flox/fl*ox^ mice. Since *Dll-1* was mutated specifically in the MuSCs and not in the myofibers, these results demonstrated definitively that DLL1 produced by committed myoblasts maintains the MuSCs in a classical lateral inhibition manner, as the authors comment [31]. Although the MuSC-myoblast interaction between receptor and ligand seems to be predominant, it is not the only one observed. A unique example of Notch/DLL1 interaction between heterologous cell types has been uncovered during embryonic myogenesis in the developing chick. In this system, Notch is transiently activated in a subpopulation of muscle cells at the epaxial lip of the dermomyotome, by DLL1^+^ progenitors migrating neural crest cells [62].

The cellular arrangement in adult, resting muscle, however, is strikingly different from the growing (embryonic and regenerating) muscle, and so are the Notch factors involved. In resting muscle, MuSCs are positioned between the basement membrane and the myofiber, the sole cell with which there is known physical contact. Hence, based on the nature of Notch signaling, the myofiber is the strongest candidate for the source of Notch ligands. Yet, ECs have drawn special attention as an alternative source of ligand, since MuSCs are closely associated to capillaries in human and mouse muscle [63, 64]. In a recent study, in silico analysis of gene expression datasets led to the hypothesis that DLL4 from ECs interact with NOTCH1 and NOTCH3 on MuSCs, through the basal lamina, to maintain the latter in a quiescent state [64]. However, in vivo experimental evidence would be needed to corroborate this intriguing theory. To date, no apparent physical contact has been demonstrated between MuSCs and ECs, a prerequisite for Notch signaling-across the basal membrane, and soluble forms of Notch ligands have not been shown to exist or be active in vivo.

Despite the suggestions of alternative sources, several studies point to the myofibers as the major and essential source of ligands. In fetal chick myogenesis, Notch activation in MuSCs is achieved by muscle contractions that force the myonuclear localization of the mechanosensory transcription factor YAP and thus the expression of the Notch ligand JAG2 [65]. In mice, an intriguing study showed that circulating sex hormones enhance the presentation of DLL1 and JAG1 ligands in the membrane of newly formed myofibers during puberty, by modulating the activity of the E3 ubiquitin ligase Mindbomb 1 (Mib1) [27] (Fig. 2A). Of interest, inhibition of sex hormones by orchiectomy significantly reduced the number of self-renewed quiescent PAX7^+^ cells during puberty and muscle regeneration but had no impact on the maintenance of the quiescent MuSC in homeostatic muscles. One possible explanation for this distinction is that sex hormones impact Mib1 only in fresh myotubes and not adult myofibers. Alternatively, sex hormones might regulate Notch ligand presentation in other cell types, which are in contact with the MuSC only during growth and regeneration, but not in resting muscles. A third explanation could be that other ligands are dominant to maintain quiescent MuSCs. In fact, it is now established that DLL4 is expressed in newly formed myotubes and mature myofibers [32, 66, 67] in an MIB1-dependant manner, and it is required for the maintenance of quiescent MuSCs. The original investigation using primary cell culture models [66] was later confirmed in vivo using conditional *Dll-4* flox deletion in muscle fibers (tamoxifen-inducible *HSA*^*CreMER*^; *Dll4*^*floxflox*^ mice) [32]. Thirty days after injecting tamoxifen for seven consecutive days resulted in a 50% decrease in the total number of PAX7^+^ MuSCs in *Dll-4* cKO fibers compared to controls. It remains to be shown if the remaining MuSCs are eventually depleted at a later time point, or whether they are present due to an alternative ligand or partial recombination efficiency.

### Oscillatory signal propagation during MuSC self-renewal

The results discussed above made it seem as if it was “problem solved” for Notch-ligand regulation in muscle stem cells. We know that there is a high level of Notch receptors and activity in stem cells, with MuSCs exhibiting Notch1, Notch2, and Notch3 receptors and high levels of *Hey1*, *HeyL*, and *Hes1*, and that this stem cell pool diminishes as cells differentiate and express MYOD to induce *Dll-1* [31, 68, 69]. This mechanism of receptor/ligand regulation is highly conserved and utilized in other cell types such as radial glia cells, fly sensory organ receptors and intestinal stem cells to name a few [70–72]. Yet, the interplay between Notch signaling and myogenic commitment has turned out to be more dynamic than previously thought. Elegant work from Lahmann et al. demonstrated that HES1 drives oscillatory *Myod* expression by binding to upstream regulatory and promoter sequences of the *Myod* locus [34]. In this case, oscillatory MYOD is not acting as a differentiation factor but in fact sustains the cells in a proliferative state. Consistently, ablation of *Hes1* resulted in irregular *Myod* oscillations and drove cells towards differentiation. Later, Zhang and colleagues elaborated on these findings and linked oscillatory *Dll-1* activity to *Hes1* expression, demonstrating that the oscillatory dynamics of *Dll-1* are critical for maintaining the proper balance between self-renewal and differentiation of MuSCs [31]. It would be interesting to study this oscillation network in the context of overload-induced hypertrophy where the majority of MuSCs enter the cell cycle but do not seem to express *Myod* [47]. Moreover, another element in the balance between proliferation and differentiation is the cell-cycle inhibitor p57. Antagonistic binding to p57 enhancers between the HES1/HEY1 repressors and the MYOD activator was shown to ensure the sustained proliferation of MuSCs [73]. Notably, the mechanism of p57 regulation established in the embryonic myotome is likely to be conserved in the regenerating muscle, where p57 in linked with the differentiated MYOG^+^ cells [74] (Fig. 2A).

### Notch regulated myogenesis in invertebrates

In the roundworm *C. elegans*, muscle cells arise from the two blastomeres, AB and predominantly P1, that result from the first division of the fertilized egg. Both striated and non-striated muscles comprise the musculature of this animal, but the obliquely striated muscles known as striated body wall muscles are the functional equivalent of vertebrate skeletal muscles [75, 76]. Adult *C. elegans* have 95 body wall muscles cells, 81 of which are specified embryonically and the remaining 14 post-embryonically. Despite their divergent cell ancestors, Notch signaling is an integral component for the specification of both embryonic and post-embryonic muscle cells [77].Additional studies on *lin-12* and *glp-1* (the two Notch receptor orthologues in *C. elegans*) double mutant embryos found an absence of the anal depressor muscle and at least one intestinal muscle. 6 of the 14 body wall muscles become dorsal, while the remaining 8 join the ventral quadrants. In the ventral cells, loss of *Lin-12*/Notch signaling results in a ventral-to-dorsal fate transformation [78] as this pathway normally functions to promote the ventral sex myoblast fate [79]. It therefore becomes evident that in *C. elegans* Notch acts in different stages and lineages of muscle development.

In the *Drosophila* embryo, Notch/Delta-mediated lateral inhibition restricts the expression of the proneural gene *lethal of scute* in a precise cell that will become the myogenic progenitor [80]. Therefore, early loss of Notch function causes hyperplasia of muscle progenitor cells in the mesodermal layer [81]. At mid-embryogenesis (stage 12), the adult muscle precursors (AMPs) emerge from the muscle progenitors by asymmetric segregation of the Notch signaling antagonist *Numb* [82]. Consistently, Notch signaling is active in embryonic AMPs, as assessed by the expression of the target gene *Enhancer of split m6* [80]. In contrast to vertebrate myogenesis, AMPs are quiescent during embryonic and most of larval life and enter the cell cycle at the second instar larva stage to ensure adult muscle growth and the regeneration of a subset of thoracic flight muscles. Targeted expression of NICD in embryonic quiescent AMPs does not alter their number, however, in third-instar larvae, where the AMPs are proliferating, it induces significantly higher numbers of AMPs [83]. An ostensibly opposite observation has been seen in the mouse where sustained Notch signaling in muscle progenitors (*Myf5*^*Cre*^*-NICD*) increases the number of MuSCs during embryogenesis, whereas in the adult, NICD overexpression has no impact on the size of the quiescent pool [23, 84]. However, considering the significant developmental differences between the two organisms, Notch seems to have an evolutionarily conserved role in respect to the cell state to promote proliferating progenitors and maintain quiescent stem cells.

### Notch modifiers operating in MuSCs

A protein linked to Notch signaling in skeletal muscle cells that has drawn attention is MEGF10, mutations in which cause a rare recessive congenital myopathy, as discussed later [85]. In resting muscle, this multiple epidermal growth factor repeat transmembrane protein is specifically expressed in quiescent MuSCs and its expression is maintained in the proliferating myoblasts but not in terminally differentiated myofibers [86]. Knock down of *Megf10* closely resembles Notch loss-of-function phenotypes in MuSCs, including ectopic Myogenin expression and precocious differentiation [86]. Additionally, tissue culture overexpression systems have shown binding between the intracellular domains of MEGF10 and NOTCH1, yet the actual physical and functional interactions of the endogenous proteins remain unclear [87].

Another critical factor for MuSC maintenance is the dual-specificity lipid and protein phosphatase PTEN. Conditional deletion of this tumor suppressor gene leads to gradual depletion of quiescent MuSCs, similar to the loss of the Notch pathway effector *Rbpj*. Mechanistically, *Pten* deletion increases AKT phosphorylation, which induces cytoplasmic translocation of FOXO1 and suppression of Notch signaling since FOXO1 binds nuclear NICD and acts as co-activator [37]. Another member of the forkhead box O family of transcription factors, FOXO3, has been reported to regulate MuSC self-renewal, but not quiescence, in a putative Notch-related manner [40]. Interestingly, *Pten*-null MuSCs spontaneously exit quiescence and undergo terminal differentiation with S-phase entry but bypassing proliferation. This is analogous yet distinct from the *Rbpj* cKO MuSCs that spontaneously differentiate without reaching the S-phase [14, 18]. Of note, in *Drosophila* AMPs PTEN has evolved an antagonistic relationship with Notch signaling. There, insulin signaling triggers Notch activation to drive proliferation of AMPs [88]. Consequently, since PTEN is a negative regulator of Insulin/PI3K signaling, PTEN overexpression in AMPs phenocopies Notch-RNAi overexpression [88].

A modifier of Notch that is worth mentioning is the basic helix-loop-helix transcription factor STRA13. Although dispensable for skeletal muscle development, *Stra13* KO mice show strong regeneration defects (small degenerating myofibers and increased fibrosis) and elevated Notch activity. Overexpression experiments in culture suggest that STRA13 is physically interacting with the intracellular domain of NOTCH1, antagonizing its binding to RBPJ [89]. Moreover, an interesting case of paracrine signaling comes from the interstitial macrophages that express the metalloproteinase ADAMTS1 [90]. Ectodomain shedding metalloproteinases, like ADAM10 and ADAM17, are critical for Notch receptor activation (proteolytic S2 cleavage of the extracellular domain). Conditional deletion of *Adam10* in MuSCs results in severe regeneration defects due loss of MuSCs [39]. On the contrary, overexpressed ADAMTS1 in macrophages was found to antagonize Notch signaling in MuSCs during regeneration, likely by interfering with the S2 cleavage of NOTCH1 [90].

Additional Notch pathway regulation is exerted at the level of RNA translation by the methyltransferase METTL3 [38]. METTL3 catalyzes the N6-methyladenosine RNA modification, the most abundant post-translational mRNA modification, with strong effects on mRNA stability, splicing, and translation. By performing MeRIP-Seq (methylated RNA immunoprecipitation and sequencing) in C2C12 cells, the authors identified many METTL3 targets, including Notch pathway components JAG1, NOTCH2, RBPJ, and MAML1. Of interest, it was shown that METTL3 regulates these transcripts at the translation level and has no effect on transcription. Mice with conditional deletion of the *Mettl3* flox allele in MuSCs show defective regeneration with reduced proliferating PAX7 cells. On the other hand, no defect was observed in quiescent mutant MuSCs, a phenotype that is not consistent with a role of METTL3 as an agonist of several Notch components [14, 18].

The regulation of MuSCs by Notch signaling is also tightly linked to their microenvironment. During aging and in the absence of sufficient niche support, the fact that activated MuSCs become susceptible to cell death has been mechanistically linked to Notch signaling. Interestingly, it was shown that the Notch target HEY1 directly binds to a consensus E-box sequence in the promoter of *Mdm2* and suppresses its expression, thus stabilizing p53 and preventing MuSC death [91] (Fig. 2B). Moreover, by direct transcriptional control, Notch has been shown to control MuSC adhesion to the niche and the composition of its extracellular matrix (ECM). The NICD/RBPJ transcriptional complex binds and induces the regulatory elements of microRNA-708, which is highly expressed in quiescent cells and sharply downregulated in activated cells [11, 36, 92]. Functional studies show that miR-708 regulates quiescence and self-renewal by antagonizing cell migration through targeting the transcripts of the focal adhesion-associated protein Tensin3 [36] (Fig. 2B). Therefore, this study demonstrates that dynamic regulation of the migratory machinery is an additional means by which Notch signaling regulates MuSC quiescence and transition to activation. Also, using mouse genetic models, it was shown that during development, the Notch pathway is critical for the colonization of the MuSC niche by directly controlling expression of cell adhesion and ECM proteins [26]. Subsequently, these observations were expanded to the adult, homeostatic tissue. There, it was found that the NICD/RBPJ transcriptional complex induces genes encoding for the collagen type V (COLV) trimeric complex. In this context, Notch, by controlling the production of COLV through the MuSC, puts in place a cell-autonomous system of regulation whereby COLV binds and activates the G-protein coupled receptor CalcR (calcitonin receptor), also produced by the MuSCs (Fig. 2B) [35]. Therefore, Notch regulates MuSCs by antagonizing differentiation, sustaining an oscillatory network, enhancing anchorage, and by shaping their microenvironment.

### Notch dysregulation and myopathies

Several genetic mutations have been identified in congenital myopathies that are characterized by skeletal muscle weakness and lack of muscle tone. Given the central role of Notch signaling in myogenesis, it is not surprising that mutations in members of this pathway have been linked to diverse myopathies. The genes identified are modifiers of Notch signaling or ligands of the Notch receptor, but no mutations have been found in the loci encoding for any of the four Notch paralogues. It is also worth considering that since Notch is acting on the stem and progenitor cells, but not the differentiated myofibers, the Notch-linked myopathies possibly have their root in dysregulated MuSCs, a group of myopathies recently defined as satellite cell-opathies [93].

A study following 4 patients that demonstrate an autosomal recessive limb-girdle muscular dystrophy (LGMD), points to the missense mutation D233E on *POGLUT-1* causing Notch signaling reduction, as the primary cause of the disease manifestation [94]. Studies in flies have shown that the O-glycosyltransferase-1 gene *POGLUT-1* promotes Notch signaling by adding O-glucose on EGF repeats that harbor specific conserved sequences, as the ones found in the extracellular domain of NOTCH1 [95]. The patients identified showed normal POGLUT-1 mRNA and protein levels. However, in vitro studies using purified D233E human POGLUT-1 uncovered reduced enzymatic activity toward 5 different EGF repeats of mouse *Notch1*. Additionally, immunohistochemistry on muscle sections and studies using D233E primary myoblasts found significant reduction in MuSCs, proliferating myoblasts, and fusion when compared to healthy controls. A follow up study by the same group, identified additional POGLUT-1 mutations in LGMD patients, substantiating the leading role of POGLUT-1 in a subset of LGMDs [96]. Furthermore, experiments on C2C12 murine cells treated with gamma secretase inhibitors, showed reduction of glycosylated alpha-dystroglycan, a common feature of LGMD, further supporting the idea of aberrant Notch signaling as the leading cause for these LGMD cases [96].

A rare, recessive congenital severe myopathy described as early-onset myopathy, areflexia, respiratory distress, and dysphagia (EMARDD) is associated with mutations in the *MEGF10* locus [97]. SNP mapping in one patient identified a 10-bp duplication in a *MEGF10* exon that caused a frameshift mutation and predicted a null allele. Additional studies led to the identification of similar nonsense *MEGF10* mutations in other patients previously described as spinal muscular atrophy with respiratory distress (SMARD) cases. In the following years, several independent studies demonstrated the role of MEGF10 in other EMARDD patients with a typical EMARDD or milder phenotype that were characterized by similar frameshift mutations [85, 98, 99]. The suggested link of *Megf10* with Notch signaling raises the possibility for an underlying Notch involvement in this relatively recently characterized myopathy.

Other studies on patients with muscle disorders show that abrogated Notch signaling can be the cause of muscular diseases due to mutations in Notch ligands. In a more recent study, affected individuals harbored homozygous or heterozygous variants of *JAG2*. Bioinformatic tools predicted that these mutations may be damaging for the protein, but functional data is missing [100]. An experimental setup where the patients’ *JAG2* point mutations could be explored in terms of Notch target activation, would be informative.

Evidence for an involvement of Notch signaling in the onset of muscular diseases was further provided in a study on Golden Retriever muscular dystrophy (GRMD) in dogs. GRMD dogs are characterized by a complete absence of dystrophin and provide a severe preclinical model of Duchenne muscular dystrophy (DMD). Two unique cases of GRMD dogs, however, have been identified that were only mildly affected by the absence of Dystrophin [101]. Extensive whole genome studies revealed an SNP in the promoter region of *Jag1* that created a novel myogenin binding site. In vitro luciferase assays using the variant sequence showed specific binding by myogenin, whereas *Jag1* expression was significantly increased in HEK293T cells overexpressing myogenin upon transfection with the variant vector. Furthermore, overexpression of *Jag1* in the *sapje* zebrafish dystrophic model revealed a fiber organization that resembled that of the WT zebrafish. Proliferation assays revealed that canine MuSCs harboring this SNP retain a robust proliferation capacity in vitro, comparable to that of MuSCs from WT dogs, and in contrast to those from severely affected GRMD. Of note, unlike *Dll-1*, *Jag1* is highly expressed in MuSCs and its expression is reduced with differentiation. It remains unclear how the ectopic expression of this Notch ligand can rescue the DMD phenotype. As the authors speculate, it is likely that JAG1 is the main mediator of the regenerative process that is disrupted by dystrophin-deficient muscles.

Undoubtedly, numerous studies in the MuSC field show that Notch is a key player in MuSCs in development and regeneration and its emerging role in muscular diseases could lead to the discovery of novel therapeutic targets.

## Conclusions: notch, a multifunctional signaling network

Extensive work has been performed on receptors, ligands, and their modifiers in Notch signaling, whereas insight into the downstream targets are mainly limited to the Hes/Hey-mediated repression of myogenic factors. Consequently, the major role that has emerged for Notch signaling on MuSC maintenance is to antagonize differentiation. This function is well conserved across species in muscles of diverse developmental origins (trunk and head muscles) and is essential for two opposite cells states: proliferation (self-renewal) and mitotic quiescence. In resting muscle, receptor-bearing, quiescent MuSCs are in direct contact with the cell-free basal lamina and the ligand-bearing myofiber, securing a tight system of directional Notch signaling. In this context, Notch has evolved to control MuSC anchoring and build-up of the ECM niche. Of note, these parallel pathways controlled by Notch are not redundant, as inactivation of either one independently leads to exit from quiescence and diminution of the MuSC pool (antagomirs against miR-708 [36]; *Hey1/HeyL* double knock-out [16] and *Col5a1* [35] or CalcR knock-outs [102]). Taken together, as described in this review, the range of transcriptional targets and modifiers has significantly expanded in recent years, introducing new functions regulated by Notch, thus constituting a complex intertwined network rather than a single linear pathway.

## Data Availability

Not applicable.

## Abbreviations

LAR: Leukocyte-common Antigen-related Receptor tyrosine phosphatase
AMP: Adult muscle precursor
APP: Amyloid precursor protein
C. elegans: Caenorhabditis elegans
cKO: Conditional knock out
COLV: Collagen type V
CSL: CBF1, Suppressor of Hairless, Lag-1
Dll1: Delta-like-1
DMD: Duchenne muscular dystrophy
dpi: Days post-injury
E-box: Enhancer-box
EC: Endothelial cell
ECM: Extracellular matrix
EMARDD: Early-Onset, Myopathy, Areflexia, Respiratory Distress, And Dysphagia
GRMD: Golden retriever muscular dystrophy
HEK293T: Human embryonic kidney 293t
KO: Knock out
LGMD: Limb-girdle muscular dystrophy
MAML-1: Mastermind-like 1
MYOG: Myogenin
Mef2C: Myocyte enhancer factor 2C
MeRIP-Seq: Methylated RNA immunoprecipitation and sequencing
Mib1: Mindbomb 1
miR-708: MicroRNA-708
MuSCs: Muscle stem cells
Mymk: Myomaker
NICD: Notch intracellular domain
RBPJ: Recombination signal binding protein for immunoglobulin kappa J region
SMARD: Spinal muscular atrophy with respiratory distress
SNP: Single-nucleotide polymorphism
Su(H): Suppressor of hairless protein
WT: Wild type
